# Doing It Your Way: How Individual Movement Styles Affect Action Prediction

**DOI:** 10.1371/journal.pone.0165297

**Published:** 2016-10-25

**Authors:** Atesh Koul, Andrea Cavallo, Caterina Ansuini, Cristina Becchio

**Affiliations:** 1 C’MON Unit, Fondazione Istituto Italiano di Tecnologia, Center for Human Technologies, Via E. Melen 83, 16152, Genova, Italy; 2 Department of Psychology, University of Torino, Via Po 14, 10123, Torino, Italy; Universitatsklinikum Tubingen, GERMANY

## Abstract

Individuals show significant variations in performing a motor act. Previous studies in the action observation literature have largely ignored this ubiquitous, if often unwanted, characteristic of motor performance, assuming movement patterns to be highly similar across repetitions and individuals. In the present study, we examined the possibility that individual variations in motor style directly influence the ability to understand and predict others’ actions. To this end, we first recorded grasping movements performed with different intents and used a two-step cluster analysis to identify quantitatively ‘clusters’ of movements performed with similar movement styles (Experiment 1). Next, using videos of the same movements, we proceeded to examine the influence of these styles on the ability to judge intention from action observation (Experiments 2 and 3). We found that motor styles directly influenced observers’ ability to ‘read’ others’ intention, with some styles always being less ‘readable’ than others. These results provide experimental support for the significance of motor variability for action prediction, suggesting that the ability to predict what another person is likely to do next directly depends on her individual movement style.

## Introduction

Understanding others’ intentions is a prerequisite for successful social interaction [1]. But how do we understand the intentions of other people? Is it possible to understand the intentions of others by simply observing their movements? Few studies have directly assessed the ability to judge intention from movement and results have been mixed [2,3]. Some studies report observers to be able to discriminate intentions from the observation of early differences in movement kinematics [4,5]. In keeping with this notion, for example, [4] showed that, by simply observing grasping movements, observers were able to judge whether the agent’s intent in grasping the object was to cooperate with a partner or compete against an opponent. Other studies, however, did not confirm such advance information pickup from others’ grasping patterns. For example, [6] found that observers were not able to predict whether an object was going to be brought to the mouth or placed on the table until they had seen at least part of the post-grasp movement—a finding that was taken to suggest that they did not detect early kinematic differences to predict the outcome of observed actions.

Central to being able to interpret these apparently contradictory results is the understanding of the relationship between the specific features of observed movements and the capacity to discriminate intention. Prior studies have largely ignored variations in motor performance, assuming movement features to be similar across trials and individuals. Repeatedly performing a movement, however, does not result in the same motor output on every attempt. More importantly, in performing the same task, outputs of the motor system may vary substantially from one individual to another [7]. Individual variations in movement patterns have been documented in a wide variety of animals [8], as well in humans [9]. For example, individuals have been shown to use their own unique set of motor synergies, which varies in both structure and number, but is consistent across different motor tasks and over a period of weeks [10]. While differences in anatomy may contribute to such variations, it is likely that motor exploration, experience, and training also influence subject-specific muscle strategies, leading to dissimilar kinematic patters for achieving an equivalent goal.

One issue that research on action observation has not considered until now is whether differences in movement style influence action understanding. Some movement styles may facilitate understanding of others’ intention, whereas others may make it more difficult. If so, the mixed results regarding intention-from-movement understanding may reflect, at least in part, differences in the individual style of the observed movements.

To examine this possibility, in the present study, we first recorded grasping movements performed with different intents and used a two-step cluster analysis to identify quantitatively ‘clusters’ of movements performed with similar movement styles (Experiment 1). Next, using videos of the same grasping movements, we probed observers’ ability to judge intention from the observation of different movement clusters (Experiments 2 and 3). To briefly preempt our results, we found that the capacity to discriminate intention was modulated by the individual style of the observed movement such that, across different tasks, some movement styles were always less ‘readable’ than others were. These findings provide the first demonstration that individuality—regarded as a fundamental neuromechanical principle of how the motor system plans and learns movements—also influences action observation.

## Experiment 1: Action Execution—Methods

### Participants

Seventeen participants (9 females, mean age 28.17 years, range 21–39) were recruited for the experiment. All participants were right handed, had normal or normal to corrected vision, and no history of neurological disorders. The research study was approved by local ethical committee (Comitato Etico Regione Liguria) and was carried out in accordance with the principles of the revised Helsinki Declaration (World Medical Association General Assembly, 2008). All the participants provided written informed consent.

### Apparatus and Procedure

The participants were seated on a height-adjustable chair with the elbow and wrist resting on a table (length = 110 cm; width = 100 cm). In order to guarantee a repeatable start position across participants, they were asked to maintain the forearm pronated, the right arm oriented in the parasagittal plane passing through the shoulder, and the right hand in a semi-pronated position, with the tips of the thumb and index finger on a tape-marked point placed on the working space. A glass bottle was positioned on the table at a distance of about 46 cm from participants’ body midline. Depending on condition, participants were instructed to reach towards and grasp the bottle with one of the following intentions:

1. *grasp-to-pour*: grasp the bottle to pour some water into a small glass (diameter: 5 cm; height: 8.5 cm) positioned on the left side of the bottle (at a distance of 25 cm);
2. *grasp-to-place*: grasp the bottle and place it in a cardboard box (length: 17 cm; width: 17 cm; height 12.5 cm) positioned on the left side of the bottle (at a distance of 25 cm);
3. *grasp-to-drink*: grasp the bottle to drink some water from it.

The immediate spatial demands and the accuracy requirements were kept constant for each intention across trials. The experimenter visually monitored the performance for each trial to ensure participants’ compliance to task requirements. Participants performed two sessions of 30 trials each, in 3 separate blocks of 10 trials for a total of 2 blocks of 10 trials for each of the three intentions. The blocks were pseudo-randomized to ensure that the last block of the first session was different from the first block of the second session. The first session was preceded by a practice session to familiarize participants with the task (12 practice trials; 4 trials for each intention).

### Kinematics and Video Recording

A near-infrared camera motion capture system (frame rate, 100 Hz; Vicon System) equipped with nine cameras was used to track the hand kinematics. Each participant was outfitted with lightweight retro-reflective hemispheric markers (4 mm in diameter) placed on the radial aspect of the wrist, the metacarpal joint and the tip of the index finger, the metacarpal joint of the little finger, the trapezium bone of the thumb, and the tip of the thumb. Movements were also filmed from a lateral viewpoint using a digital video camera (Sony Handy Cam 3-D, 25 frames/sec). The video camera was placed at about 120 cm from participant’s hand starting position with the camera view angle directed perpendicularly to the agent’s midline. Video camera position and arrangement were kept constant for the entire duration of the study in order to ensure that only the hand and the bottle were in full view from the beginning up to the end of the movement.

### Kinematics Data Processing

After data collection, each trial was individually inspected for correct marker identification and then run through a low-pass Butterworth filter with a 6 Hz cutoff. Kinematics variables of interest were computed for the reach-to-grasp phase of the movement, defined as the phase from reach onset (i.e. the first time at which the wrist velocity crossed a 20 mm/s threshold) to grasp offset (i.e. the time at which the wrist velocity dropped below a 20 mm/s threshold). Within this time window, a custom software (Matlab; MathWorks, Natick, MA) was used to compute two sets of variables: F_global_ and F_local_ variables. F_global_ variables were expressed with respect to the global frame of reference, i.e., the frame of reference of the motion capture system. Within this frame of reference, we computed the following variables:

- *wrist velocity*, defined as the module of the velocity of the wrist marker (mm/sec);
- *wrist height*, defined as the z-component of the wrist marker (mm);
- *wrist horizontal trajectory*, defined as the x-component of the wrist marker (mm);
- *grip aperture*, defined as the distance between the marker placed on thumb tip and the one placed on the tip of the index finger (mm).

To provide a better characterization of the hand joint movements, the second set of variables was expressed with respect to a local frame of reference centered on the hand (i.e., F_local_; see [11] for a detailed description of the F_local_). Within F_local_, we computed the following variables:

- *x-*, *y-*, *and z-thumb*, defined as the x-, y- and z-coordinates for the thumb with respect to F_local_ (mm);
- *x-*, *y-*, *and z-index*, defined as the x-, y- and z-coordinates for the index with respect to F_local_ (mm);
- *x-*, *y-*, *and z-finger plane*, defined as the x-, y- and z-components of the thumb-index plane, i.e., the three-dimensional components of the vector that is orthogonal to the plane, providing information about the abduction/adduction movement of the thumb and index finger irrespective of the effects of wrist rotation and of finger flexion/extension;
- *x-*, *y-*, *and z-dorsum plane*, defined as the x-, y- and z-components of the radius-phalanx plane, providing information about the abduction, adduction and rotation of the hand dorsum irrespective of the effects of wrist rotation.

All kinematics variables were expressed with respect to normalized (%) rather than absolute (ms) movement durations. After normalizing the duration of each reaching-grasping movement, the data were resampled at intervals of 0.1 of the normalized movement time. This procedure was applied to allow comparison of hand postures across trials and participants.

### Clustering parameters

To avoid incorporating the effect of intersession variability for clustering, we selected correct trials i.e. trials with correct marker identification, only from the second experimental session (grasp-to-pour = 147 trials, grasp-to-drink = 154 trials, grasp-to-place = 160 trials). The resulting kinematic parameters for these trials were normalized and submitted to ‘two step clustering procedure’ as implemented in SPSS (version 22) [12] separately for each intention. The two step clustering procedure is a time efficient, agglomerative hierarchical clustering approach that performs clustering in two steps:

1. a pre-cluster step that clusters cases into many sub-clusters;
2. a cluster step that clusters the sub-clusters resulting from pre-cluster step into the desired number of clusters.

The clustering in this procedure is based on the ‘BIRCH’ algorithm [13]. Briefly, the algorithm creates a Cluster Feature Tree (CF) that, instead of storing the actual values, stores the statistics: Number of points (N), Linear Sum (LS), and Square Sum (SS) for all clusters. These features have enough information to calculate the intra-cluster distances. This algorithm has also been shown to be relatively stable both to the input order as well as to selected parameters [13–15]. That is, unlike k-means algorithm or CLARANS, results do not vary based on the order of data points or some initial parameter setting thus, producing stable clusters.

Utilizing this clustering procedure, we first determined, separately for each intention, the optimal number of clusters by calculating Bayesian information criterion (BIC) (BIC difference ratio between successive numbers of clusters less than criteria value of 0.04). This initial estimate of the number of clusters was then refined by finding the largest relative increase in inter-cluster distance between the two closest clusters in each hierarchical clustering stage. Finally, we performed the clustering utilizing the optimal number of clusters obtained by the previous procedure and obtained the cluster number for each trial. The relative importance of each kinematic feature was also calculated. Predictor importance (PI) provides a measure of how well a specific variable differentiates between clusters. The PI of each variable *i* was calculated as

$${PI}_{i}\;=\;\frac{-{\text{log}}_{10}\left({sig}_{i}\right)}{\underset{\text{j}\epsilon \Omega }{\text{max}}(-{\text{log}}_{10}\left({\text{sig}}_{\text{j}})\right)}$$

where Ω denotes the set of predictor and evaluation fields, sig_i_ is the significance or p-value computed based on an F-test.

## Results

As expected, several distinct movement profiles emerged for each intention from the clustering analysis. The two-step analysis yielded three optimal clusters for grasp-to-pour movements, three optimal clusters for grasp-to-drink movements, and two optimal clusters for grasp-to-place movements, suggesting a reduced level of variability when grasping aimed at placing the bottle within the box. Cluster distribution profiles are provided for each intention in Table 1.

**Table 1:** Cluster distribution profile for different intentions in Experiment 1.

Intention	No. of clusters	Cluster distribution (in % of trials)
Grasp-to-pour	3	43.53%, 31.97%, 24.48%
Grasp-to-drink	3	30.51%, 29.87%, 39.61%
Grasp-to-place	2	70%, 30%

Optimum number of clusters and corresponding percentage of trials are shown for each intention.

The unequal distributions of movements indicates that, for a specific intention, some motor styles are more represented than others. In other words, the natural statistic of human movement makes some movement styles more probable than others. The relative importance of kinematic features in determining the cluster distribution profile for each intention is provided in S1 Fig. As illustrated in S1 Fig, the kinematic variable ‘z-dorsum plane’ scored higher on the importance measure for both grasp-to-pour and grasp-to-drink movements, whereas ‘grip aperture’ proved an important feature for grasp-to-place movements. Overall, whereas hand joint movements (computed with reference to a local frame of reference centered on the hand) were important in differentiating clusters for grasp-to-pour and grasp-to-drink movements, grip aperture played a more important role for grasp-to-place movements.

Crucially, although individual participant information was withheld from the modeling process, in all but 4 out of 461 trials (1 trial for grasp-to-pour, 2 trials for grasp-to-drink, and 1 trial for grasp-to-place), trials from the same participant (for a specific intention) were assigned to the same cluster. This indicates that variations in movement performance did not emerge from online, trial-by-trial optimization, but reflected differences in individual movement styles [7].

## Experiment 2: Action Observation

The finding that individuals have their own motor program styles, i.e., they show significant individual variations in outputs of the motor system, raises the question of whether individuality of motor style influences the ability to discriminate intention. To address this possibility, in Experiment 2 we used clustering information to establish correspondence between the style of the observed movement and the capacity to discriminate intention in a binary choice design.

## Methods

### Participants

Eighteen participants (9 females, mean 24.78 years, range 20–32) were recruited for the experiment. All participants were right handed, had normal or normal to corrected vision, and did not have any neurological disorders. All the participants gave written informed consent to participate in the experiment. None of them participated in Experiment 1.

### Stimuli

Video clips of grasp-to-pour and grasp-to-drink movement recorded in Experiment 1 served as stimuli for Experiment 2. After removing videos with technical problems (e.g., hand temporarily out of sight due to trajectory height; water reflection and movement in the bottle; n = 51), we equated the number of movements for each intention based on the intention with least number of trials (grasp-to pour, n = 128). Then, we performed linear discriminant analysis (LDA) using the kinematics variables as predictors and the intention as target and, based on LDA results, we selected, for each intention, the 50 movements that minimized the distance from their own centroid (i.e. the mean variate score for each intention). This procedure allowed us to identify, for each intention, 50 representative movements. As shown in Table 2, cluster distribution profiles (in % of trials) comprised an unequal distribution of movements per cluster similar to that obtained in Experiment 1. This suggests that the selected movements reflected the natural prevalence of movement styles over the entire distribution of movements. The corresponding videos were edited using Adobe Premiere Pro CS6 (.mp4 format, disabled audio, 25 frames/s, resolution 1280 × 800 pixel). To ensure that only advance sources of information were made available to participants as to judge the agent’s intention, video clips were temporally occluded at the time the fingers contacted the object. Each video clip started therefore with the actual reach onset and ended at grasp offset, with the duration of the video varying according to the actual duration of the movement (from 760 ms to 1640 ms). Neither the second part of the movement nor other objects on the table were visible (see sample S1 Video). The actors in the videos were all Caucasian and wore a black colored shirt. The percentage of movements performed by male and female agents was also controlled to be similar across intentions.

**Table 2:** Cluster distribution profiles for representative movements in Experiment 2 and Experiment 3.

Intention	Cluster	Cluster distribution (% of trials)	Number of actors	Number of movements per actor
Grasp-to-pour	1	44%	8	6,5,3,6,9,5,8,2
	2	33%	5	4,9,2,10,8
	3	23%	5	5,1,4,7,6
Grasp-to-drink	1	30%	5	3,3,3,2,4
	2	30%	5	1,7,3,3,1
	3	40%	7	3,3,2,1,4,2,5
Grasp-to-place	1	78%	10	5,2,4,3,5,6,2,5,4,3
	2	22%	5	1,3,4,1,2

Percentage of trials and number of actors for each cluster are shown for each intention.

### Procedure

**Fig 1:**
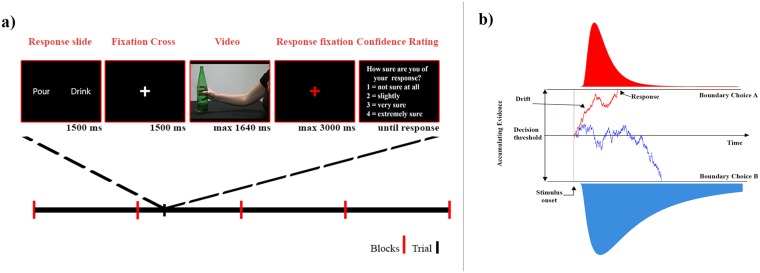
a) Experimental design for Experiments 2 and 3. Participants were exposed to 4 blocks of 100 trials (50 trials for each intention). Each trial started with the onset of two cues specifying the possible intentions. Then, a fixation cross appeared, followed by the video stimulus. Participants were free to respond either during the video presentation or in the subsequent 3000 ms. b) Representation of a drift diffusion model. The model defines parameters of rate of evidence accumulation, drift rate (‘v’), and separation between the decision boundaries, decision threshold (‘a’).

The experiment was carried out in a dimly lit room. Participants sat in front of a 17- inch computer screen (1280 x 800 resolution, 75 Hz), at a viewing distance of 50 cm. Stimuli presentation, timing, and randomization procedures were controlled using E-prime (version 2.0.10.242). Task structure conformed to a binary choice design (Fig 1a). Participants were asked to watch videos of grasp-to-pour and grasp-to-drink movements and to decide if the observed movement was performed with the intent to pour or drink. Each trial began with the presentation of a white central fixation cross (1500 ms), followed by the viewing of a grasping movement. Participant were instructed to respond as accurately and quickly as possible. They could indicate a decision at any time after reach onset by pressing one of two active buttons on a keyboard. When no response was given during the video, a red central fixation cross was displayed until response or 3000 ms had elapsed. After response, participants were also requested to rate confidence of their decision on a 4-level scale by pressing a key (data not analyzed in the current study). Each participant completed a total of 400 trials (200 trials for each intention), with 4 repetitions of each movement. The videos were pseudo-randomized over 4 blocks so that each block included one repetition of each movement. The experimental session lasted approximately 50 minutes.

To familiarize with the type of stimuli, before the beginning of the experiment, participants were presented with the action execution set up and shown some videos displaying occluded grasp-to-pour (n = 8) and grasp-to-drink movements (n = 8) followed by two unoccluded videos displaying the agent pouring the water into the glass and bringing the bottle to the mouth to drink. No response was requested from participants during this practice.

### Dependent measures and Statistical Data Analysis

We modeled the accuracies and reaction times of participants using a drift diffusion model (DDM). Drift diffusion models have been used to model behavioral data from two-choice tasks by modeling decisions based on the accumulation of evidence from the stimuli [16,17]. DDMs specifically model different components of the cognitive decision process (rate of information uptake, bias, etc.) and represent them as different parameters. Modeling of the cognitive decision process underlying the choices with DDMs is thus useful for dissociating task parameters like task difficulty (indicated by drift rate) and effect of instructions (indicated by threshold) [16,18]. The model accesses the performance of the participants in terms of measures like speed of reaching a decision boundary (drift rate, ‘v’) and distance between the two boundaries (decision threshold, ‘a’) (Fig 1b).

In this way, theoretically distinct aspects of the cognitive decision process can be separated statistically. DDMs have thus been proposed to provide a highly detailed measure of participants’ performance, deeper insights into the observed behavior and consequently, drive theoretical advances [19,20]. Indeed, a major advantage of the DDMs is the high degree of information utilization. In contrast to conventional forms of data analysis, the diffusion model incorporates response times (RTs) for correct responses and errors, as well as the ratio of correct and erroneous responses. Additionally, DDMs provide precise, unambiguous quantification of performance (compared to accuracy and reaction times) and are immune to speed-accuracy tradeoffs [18].

We used a hierarchical Bayesian approach for the estimation of these DDM parameters (drift rate ‘v’, threshold ‘a’, and non-decision time ‘t’) as implemented in the toolbox—Hierarchical drift diffusion model (HDDM) [21]. HDDM allowed us to minimize potential confounds resulting from low stimulus probabilities in some movement styles as it can recover reliable estimates even from lesser number of trials per condition [21] To evaluate HDDM model performance, we used the Deviance Information Criterion (DIC). DIC is a commonly used goodness-of-fit measure for evaluating hierarchical models [22]. We compared DIC values (lower being better) for a model allowing drift rates to vary across clusters (‘Cluster model’) with those of a ‘Null model’ where drift rate was not allowed to change between clusters (for a similar approach see [21]). Following the usual rule of thumb, a difference of more than 10 between model DIC scores was interpreted as evidence in favor of the better (i.e., lower) scoring model [23]. Model performance evaluation showed that DIC values for the ‘Cluster model’ (DIC = 21986.42) were indeed lower than those for the ‘Null model’ (DIC = 22834.92) with a difference of 848.50. This indicates that the ‘Cluster model’ provided better fit than the ‘Null model’.

We therefore tested the significance of the estimated parameters for the ‘Cluster model’. Since HDDM toolbox utilizes a Bayesian framework, significance testing can be performed directly on the posterior distribution and results can be interpreted in terms of probabilities. Thus, we analyzed the posterior distributions for different clusters to evaluate differences between them. To this end, we calculated the proportion of the posteriors in which the drift rate for one cluster was higher that than the other. A difference of more than 5% in the posterior distribution overlap (P_p|D_) was considered significant (suggesting a higher probability of difference between the conditions). We also evaluated whether cluster drift rates were significantly different from a test value of 0 to ascertain likelihood of drifting towards the correct alternative choice. A drift rate close to 0 corresponds to a process which is equally likely to move towards either of the choices, indicating a slow rate of evidence accumulation. On the contrary, a higher positive drift rate indicates a faster evidence accumulation towards the correct alternative. Since the hierarchical estimation procedure utilized violates the independence assumption, we did not analyze subject parameter estimates in a frequentistic test. Additional confirmatory analyses were also performed on accuracy and RTs separately. Results are provided in S1 File.

## Results

**Fig 2:**
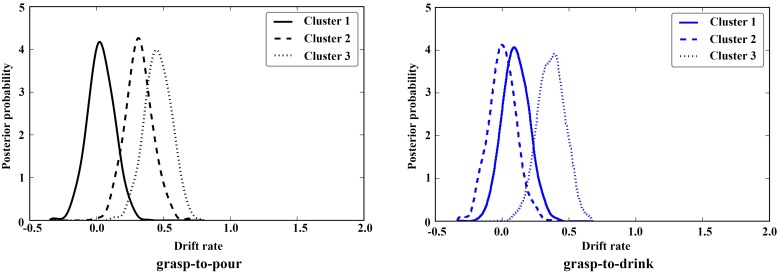
Rate of evidence accumulation (drift rate) posterior distribution densities for different clusters in Experiment 2. Clusters 1 and 2 in grasp-to-pour movements have well separated densities compared to cluster 3. In addition, cluster 3 in grasp-to-drink has significantly different distribution compared to clusters 1 and 2. Significant differences were determined when the overlap between distributions exceeded 5% (0.05).

DDM results showed significant differences between clusters on drift rates for grasp-to-pour movements. In particular, whereas similar drift rates were associated with cluster 2 and cluster 3 (posterior distribution overlap P_p|D_ [cluster 3 < cluster 2] = 0.164), drift rates for clusters 2 and 3 were higher compared to those for cluster 1 (P_p|D_ [cluster 2 < cluster 1] = 0.026; P_p|D_ [cluster 3 < cluster 1] = 0.002) (Fig 2). The difference between clusters was also significant for grasp-to-drink movements when comparing drift rates for cluster 3 to those for clusters 1 and 2 (P_p|D_ [cluster 3 < cluster 1] = 0.037, P_p|D_ [cluster 3 < cluster 2] = 0.007). No differences were found between clusters 1 and 2 (P_p|D_ [cluster 2 < cluster 1] = 0.73).

For grasp-to-pour movements, drift rates were significantly higher than 0 for cluster 2 and 3 (P_p|D_ = .001), but not for cluster 1 (P_p|D_ = 0.32) (Fig 2). For grasp-to-drink movements, drift rates were significantly higher than 0 for cluster 3 (P_p|D_ = 0.0003), but not for clusters 1 (P_p|D_ = 0.13) and 2 (P_p|D_ = 0.45).

## Experiment 3: Action Observation

Results from Experiment 2 suggest that the capacity to judge intention from motion is modulated by the individual style of the observed movements such that some movement styles (e.g. cluster 1 for grasp-to-pour movements) are less ‘readable’ than others are. The validity of this conclusion, however, may be limited to the comparison of pouring and drinking movements. In other words, it is possible that the intention to drink is harder to discriminate in cluster 1 only when the choice is between grasping movements aimed at pouring or drinking. To address this possibility, in Experiment 3 we replaced grasp-to-drink movements with grasp-to-place movements.

## Methods

### Participants

Eighteen participants (11 females, mean 26.17 years, range 22–34) were recruited for the experiment. All participants were right handed, had normal or normal to corrected vision, and did not have any neurological disorders. Written informed consent was obtained from the participants. None of them participated in Experiments 1 or 2.

### Stimuli, Procedure and Analysis

Stimulus selection and experimental task procedures were similar as those utilized in Experiment 2, except that grasp-to-place movements (duration ranging from 720 ms to 1280 ms) replaced grasp-to-drink movements (cluster distribution profiles are reported in Table 2). As in Experiment 2, we compared DIC values for a model allowing drift rates to vary across clusters (‘Cluster model’) with those of a ‘Null model’, forcing drift rate to be equal across clusters. With a DIC difference of 1137, the ‘Cluster model’ (DIC = 14651.28) was clearly better than the ‘Null model’ (DIC = 15788.75).

## Results

**Fig 3:**
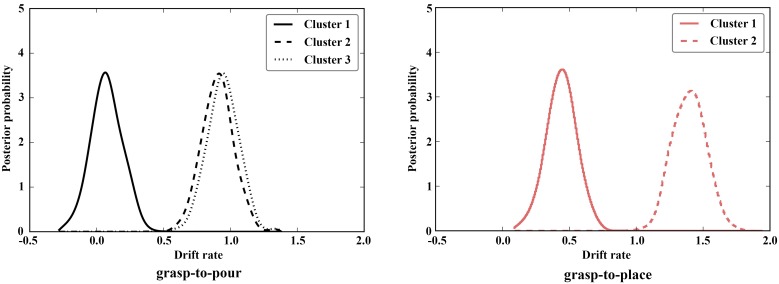
Rate of evidence accumulation (drift rate) posterior distribution densities for different clusters in Experiment 3. As in Experiment 2, clusters 1 and 2 in grasp-to-pour movements show well separated densities compared to cluster 3. For grasp-to-place, the two clusters have completely non-overlapping distributions. Significant differences were determined when the overlap between distributions exceeded 5% (0.05).

We repeated our DDM measurements on grasp-to-pour movements and found increased drift rates for clusters 3 and 2 compared to cluster 1 (posterior distribution overlap P_p|D_ [cluster 2 < cluster 1] < 0.001; P_p|D_ [cluster 3 < cluster 1] < 0.001), with no significant difference between clusters 2 and 3 (P_p|D_ [cluster 3 < cluster 2] = 0.409) (Fig 3). The pattern of results for grasp-to-pour movements was thus identical to that in Experiment 2. In addition, we also found that for grasp-to-place movements drift rates for cluster1 were lower than those for cluster 2 (P_p|D_ [cluster 1 < cluster 2] = 1.0).

As for Experiment 2, for grasp-to-pour movements, drift rates were significantly higher than 0 for clusters 2 and 3, but not for cluster 1 (P_p|D_ [cluster 1 < 0] = 0.18, P_p|D_ (cluster 2 < 0) < 0.001, P_p|D_ [cluster 3 < 0] < 0.001) (Fig 3). For grasp-to-place movements, drift rates for both clusters were significantly higher than 0 (P_p|D_ [cluster 1 < 0] <0.001, P_p|D_ [cluster 2 < 0] < 0.001).

## General Discussion

Human movements inherently incorporate variability. Previous studies in the action observation literature have largely ignored this ubiquitous, if often unwanted, characteristic of motor performance, assuming movement patterns to be highly similar across repetitions and individuals.

Here we explored whether variations in motor output influences the ability to predict others’ actions. To this end, we first quantified variations in the performance of grasping movements performed with different intents and, using two-step cluster analysis, identified ‘clusters’ of movements performed with similar styles (Experiment 1). Next, we investigated whether observers could predict the outcome of these movements as a function of movement style (Experiments 2 and 3). Our results demonstrate that individual variations in kinematic patterning directly influence the capacity to ‘read’ others’ intentions though action observation.

Individual variations in output of the motor system have been observed in walking [24] postural control [9], as well as in grasping movements [25]. For example, different movement patterns have been identified in skilled musicians, reflecting their individual history of musical training [25]. Our results add to this growing literature showing that daily grasping movements performed by untrained subjects also express variations in style. Remarkably, clustering revealed that these variations were highly consistent within a given individual, as all trials from a given subject, except four, were clustered in the same cluster. This indicates that a portion of ever-present motor variability in motor execution reflected individual differences in movement patterns, resulting in individual styles of grasping.

When we examined the implications of these findings for action observation, we found that while some styles facilitated intention discrimination, others’ styles made it more difficult. For movements in some of the clusters, discrimination was indeed not significantly above the test value of 0, suggesting an equal likelihood drifting towards either alternative (see Fig 2).

Since stimulus probabilities has been shown to influence two-choice classification [26–28], one could ask whether this pattern reflects the unequal distribution of movements per cluster. If so, we would have expected clusters associated with lower discriminability to comprise a lower number of movements. This is because low-probability stimuli are more difficult to classify than high-probability stimuli [27]. As shown in Table 2, however, this was not the case. Thus, that intention discrimination is more difficult for some styles in comparison to others does not appear to reflect stimulus probability.

This has implications for previous action observation studies in which stimuli were obtained by filming the movements performed by only a few models—sometimes a single model [29–32]. Variability in motor solutions limits the inferences that may be drawn from these studies. In case of negative findings, for example, it might be impossible to determine to what extent failure depends on the specific style of the observed movement rather than on a general inability to pick up fine kinematic information. In this regard, it is perhaps not surprising that the action observation literature does not reveal a consistent picture: our findings suggest that, in addition to task effects, these differences and inconsistencies between studies could in part result from style variations in the selected movement patterns.

It is notable that some styles appear *inherently* less readable than others. This follows from the observation that grasp-to-pour movements in cluster 1 were associated with lower drift rates both when the choice was between grasp-to-pour and grasp-to-drink movements and when it was between grasp-to-pour and grasp-to-place movements. This finding adds to the debate about whether intentions can be identified through movement observations [33]. It has been proposed that participants required to detect intention from action observation may simply compare movement profiles to detect differences in visual kinematics. On this account, rather than identify the intention associated with a specific kinematic profile (e.g., ‘grasp-to-pour’), they may thus simply detect kinematic differences between the two movements. However, if this were the case, we would expect intention discriminability patterns to change depending on the specific intention comparison (grasp-to-pour vs. grasp-to-drink or grasp-to-pour vs. grasp-to-place). This was not the case in our study. Our results rather support the proposal that intentions can *per se* be identified through the observation of others’ movements. Moreover, they demonstrate how this identifiability depends on the individual style of the observed movement.

Finally, these findings can also be considered from the perspective of theories postulating a role of the motor system in action prediction and inference [11,34]. The key concept in all these theories is that the same motor models employed during action execution also serve as the basis for action observation and prediction. In line with this, it has been shown that the accuracy of action inferences is dependent on how closely the observed movement resembles the observer’s own movement [35]. This was demonstrated by testing a heterogeneous population of individuals that included healthy subjects of different ages, as well as different movement disorder patients. The finding of individual movement styles shaping everyday actions, however, opens up the prospect for an even more fine-grained mechanism of action-perception coupling. If sensitivity to observed actions is indeed dependent upon how observers themselves execute the observed action, then commonality in motor program styles should facilitate reciprocal intention understanding. We would thus expect that the more similar the movement styles of two people are, the more effective their ability to predict each other’s actions will be.

## Conclusions

It has recently emerged as a general principle in neuromechanics that *individual*—and not averaged–solutions solve neuromotor problems when performing a motor task [7]. Averaging trials across subjects may thus obscure the underlying structure of motor outputs. Our quantitatively behavioral experiment extend the significance of this principle to action observation suggesting that *individual movement styles* directly influence the ability to understand and predict others’ actions. While certain styles facilitate action prediction, others make it more difficult. This has implication for social understanding, as it suggests that failures in social interactions between individuals may in part result from difficulties in reading intentions from certain movement styles.

## Supporting Information

S1 Dataset Dataset used for analyses in Experiment 1, Experiment 2 and Experiment 3. (XLSX)

S1 Fig Predictor importance for clustering in each intention. Top ten kinematic predictors and their respective relative importance for defining the clusters are represented. Values are shown from 0 being ‘Least Important’ to 1 as ‘Most Important’.
(TIF)

S1 File Accuracy and reaction times results for Experiment 2 and Experiment 3. Confirmatory analyses performed for Experiment 2 and Experiment 3 on accuracy and RTs separately
(PDF)

S1 Video An exemplar of reach-to-grasp stimulus used in Experiment 2 and Experiment 3. (MP4)
