# Supplementary figures and images for: Doing It Your Way: How Individual Movement Styles Affect Action Prediction
Source: PLoS One. 2016 Oct 25;11(10):e0165297. doi: 10.1371/journal.pone.0165297 (PMC5079573; doi:10.1371/journal.pone.0165297)

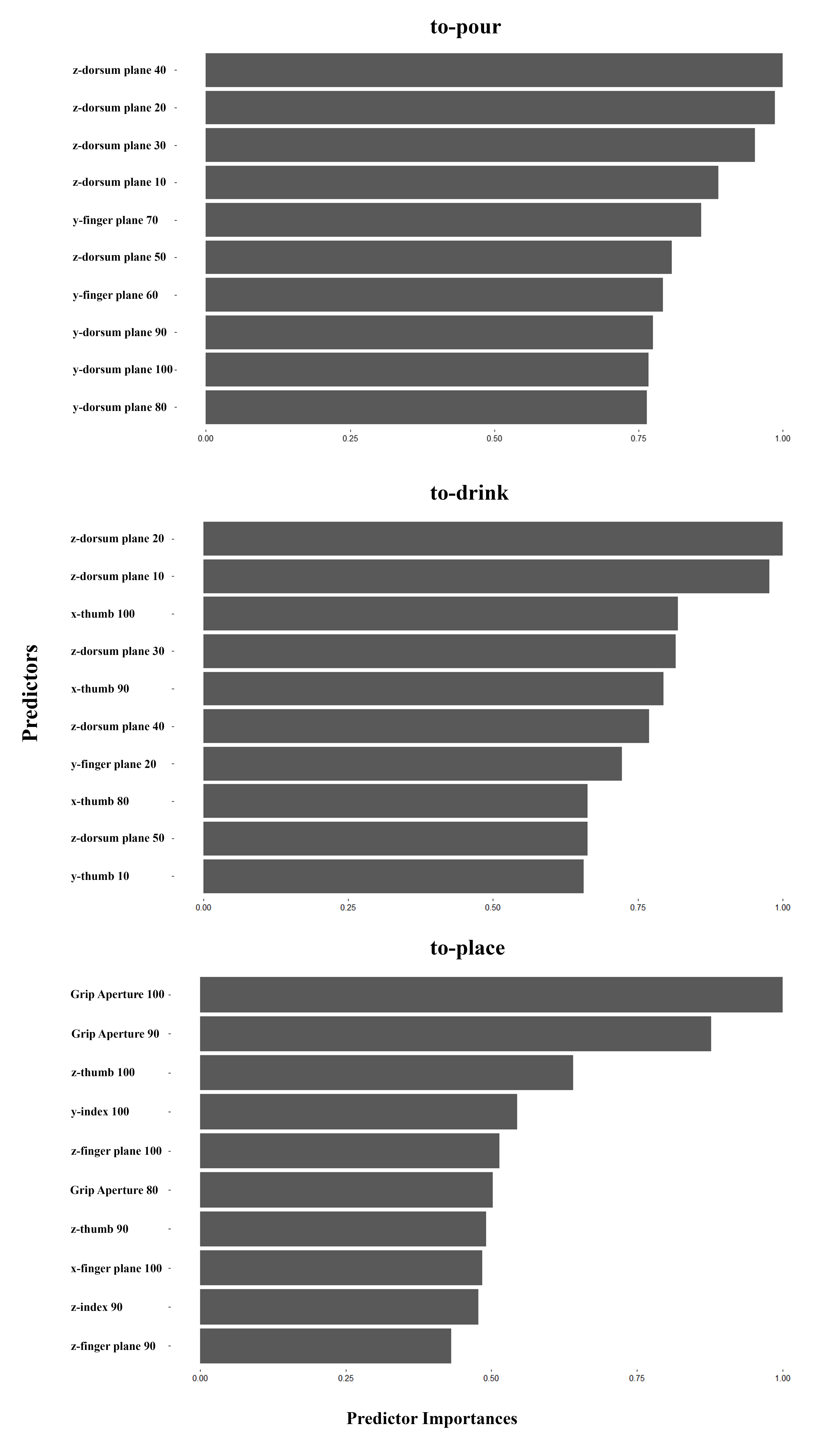

Supplement: S1 Fig — Top ten kinematic predictors and their respective relative importance for defining the clusters are represented. Values are shown from 0 being ‘Least Important’ to 1 as ‘Most Important’. (TIF) [file pone.0165297.s002.tif]
