# Supplementary material for: Doing It Your Way: How Individual Movement Styles Affect Action Prediction
Source: PLoS One. 2016 Oct 25;11(10):e0165297. doi: 10.1371/journal.pone.0165297 (PMC5079573; doi:10.1371/journal.pone.0165297)
Supplement: S1 File — Confirmatory analyses performed for Experiment 2 and Experiment 3 on accuracy and RTs separately (PDF) [file pone.0165297.s003.pdf]

Experiment 2: Action observation (grasp-to-pour vs. grasp-to-drink)

For each intention (grasp-to-pour and grasp-to-drink) *accuracy* and *normalized RTs*[1] values were submitted to a repeated-measures ANOVA with *Cluster* (1, 2, 3) as within-subjects factor. A significance threshold of $p < .05$ was set for all statistical tests and Bonferroni correction was applied for pairwise comparisons.

For grasp-to-pour movements, the ANOVA on *accuracy* values yielded a significant main effect of *Cluster* ($F_{1.52, 25.85} = 8.538$; $p = .003$, partial $\eta^2 = .334$). Post-hoc pairwise comparisons revealed that *accuracy* for Cluster 3 (mean ± SE = .723 ± .039) was significantly higher compared to Cluster 1 (mean ± SE = .514 ± .033; $p < .001$). No statistical differences were found neither between Cluster 3 and Cluster 2 (mean ± SE = .600 ± .052; $p = .071$), nor between Cluster 1 and Cluster 2 ($p = .563$) (Figure S2 panel A). The ANOVA on *normalized RTs* also yielded a significant main effect of *Cluster* ($F_{2, 34} = 37.074$; $p < .001$, partial $\eta^2 = .686$). Post-hoc pairwise comparisons showed faster RT for Cluster 2 (mean ± SE = 1.722 ± .073) compared to both Cluster 1 (mean ± SE = 1.837 ± .064; $p = .001$) and Cluster 3 (mean ± SE = 1.950 ± .074; $p < .001$). *RTs* were also faster for Cluster 1 compared to Cluster 3 movements ($p = .001$) (Figure S2 panel a).

For grasp-to-drink movements the ANOVA on *accuracy* yielded a significant main effect of *Cluster* ($F_{2, 34} = 7.452$; $p = .002$, partial $\eta^2 = .305$). Post-hoc pairwise comparisons revealed that *accuracy* for Cluster 3 (mean ± SE = .649 ± .038) was significantly higher compared to Cluster 2 (mean ± SE = .498 ± .047; $p = .012$). No statistical differences were found neither between Cluster 3 and Cluster 1 (mean ± SE = .540 ± .033; $p = .050$), nor between Cluster 1 and Cluster 2 ($p = .711$) (Figure S2 panel B). The ANOVA on *normalized RTs* also yielded a significant main effect of *Cluster* ($F_{2, 34} = 25.703$; $p < .001$, partial $\eta^2 = .602$). Post-hoc pairwise comparisons showed that for Cluster 2 (mean ± SE = 1.971 ± .086) RTs were significantly slower compared to both Cluster 1 (mean ± SE = 1.814 ± .075; $p < .001$) and Cluster 3 (mean ± SE = 1.817 ± .075; $p < .001$). No differences were found between Cluster 1 and Cluster 3 ($p = 1.000$) (Figure S2 panel b).

[1] Since video duration was different for each movement, RTs were normalized and were expressed as an ratio between the absolute RT and the video duration. Normalized RT = absolute RT / video duration

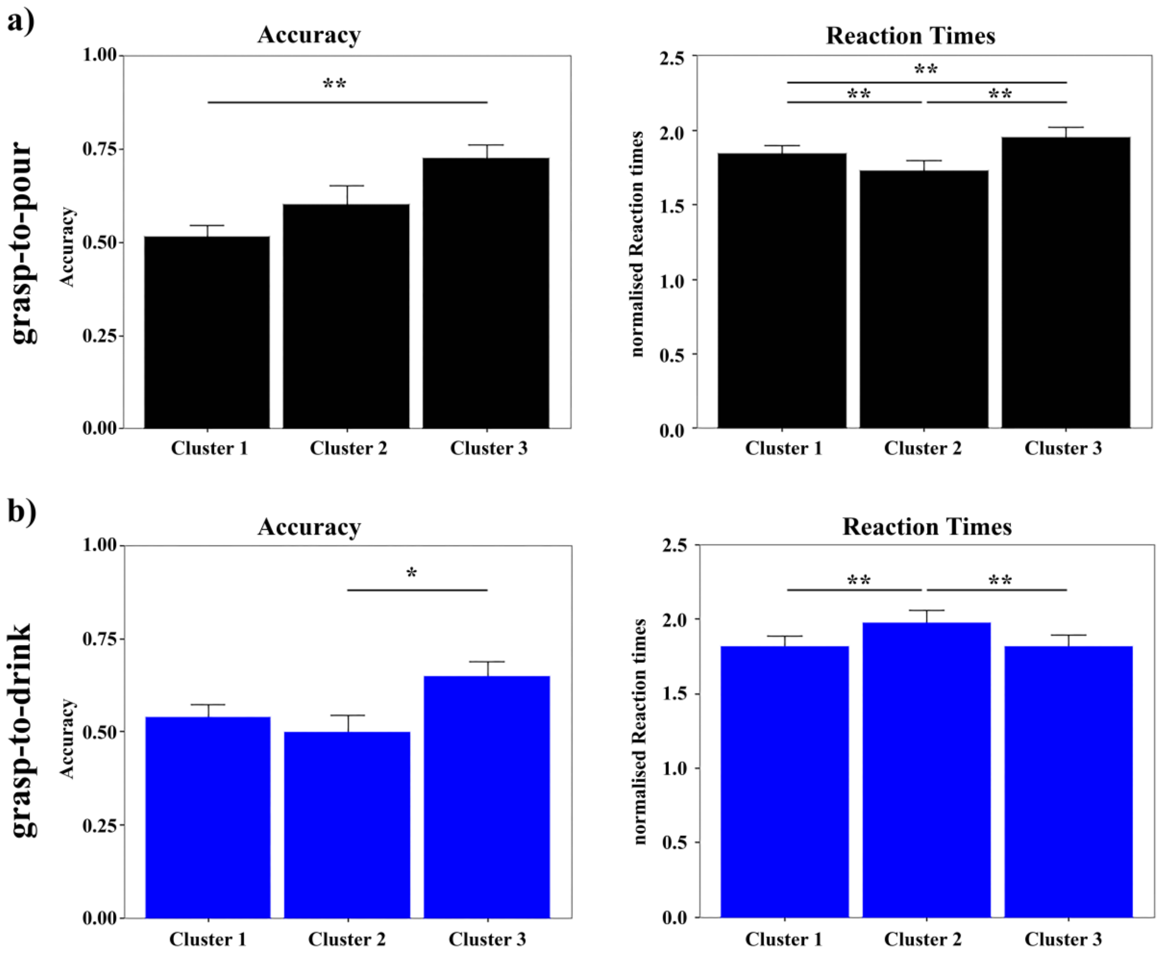

Figure S2. Accuracy and Reaction times for Experiment 2 (a) grasp-to-pour and (b) grasp-to-drink movements. (* $p < 0.05$, ** $p \le 0.001$)

Experiment 3: Action observation (grasp-to-pour vs. grasp-to-place)

For grasp-to-pour movements, *accuracy* and *normalized RTs* values were submitted to a repeated-measures ANOVA with *Cluster* (1, 2, 3) as within-subjects factor. For grasp-to-place movements paired t-tests on *accuracy* and *RTs* were performed to verify statistical differences between Cluster 1 and Cluster 2. A significance threshold of $p < .05$ was set for all statistical tests and Bonferroni correction was applied for pairwise comparisons.

For grasp-to-pour movements the ANOVA on *accuracy* values yielded a significant main effect of *Cluster* ($F_{1.29, 21.95} = 32.519$; $p < .001$, partial $\eta^2 = .657$). Post-hoc pairwise comparisons revealed that *accuracy* for Cluster 3 (mean ± SE = .854 ± .041) was significantly higher compared to both Cluster 1 (mean ± SE = .532 ± .036; $p < .001$) and Cluster 2 (mean ± SE = .761 ± .038; $p = .005$) An additional difference was found between Cluster 1 and Cluster 2 with higher accuracy in the latter case ($p < .001$) (Figure S3 panel a). The ANOVA on *normalized RTs* also yielded a significant main effect of *Cluster* ($F_{2, 34} = 17.780$; $p < .001$, partial $\eta^2 = .511$). Post-hoc pairwise comparisons showed higher RTs for Cluster 3 (mean ± SE = 1.670 ± .103) compared to both Cluster 1 (mean ± SE = 1.491 ± .079; $p < .001$) and Cluster 2 (mean ± SE = 1.500 ± .085; $p = .001$). No difference was found between Cluster 1 and Cluster 2 movements ($p = 1.000$) (Figure S3 panel a).

For grasp-to-place movements the paired t-test on *accuracy* revealed a significantly higher accuracy for Cluster 2 (mean ± SE = .874 ± .022) compared to Cluster 1 (mean ± SE = .689 ± .025; $t_{17} = 6.313$, $p < .001$). RTs for Cluster 2 (mean ± SE = 1.382 ± .082) were also significantly faster than those of Cluster 1 (mean ± SE = 1.662 ± .086; $t_{17} = 13.029$, $p < .001$) (Figure S3 panel b).

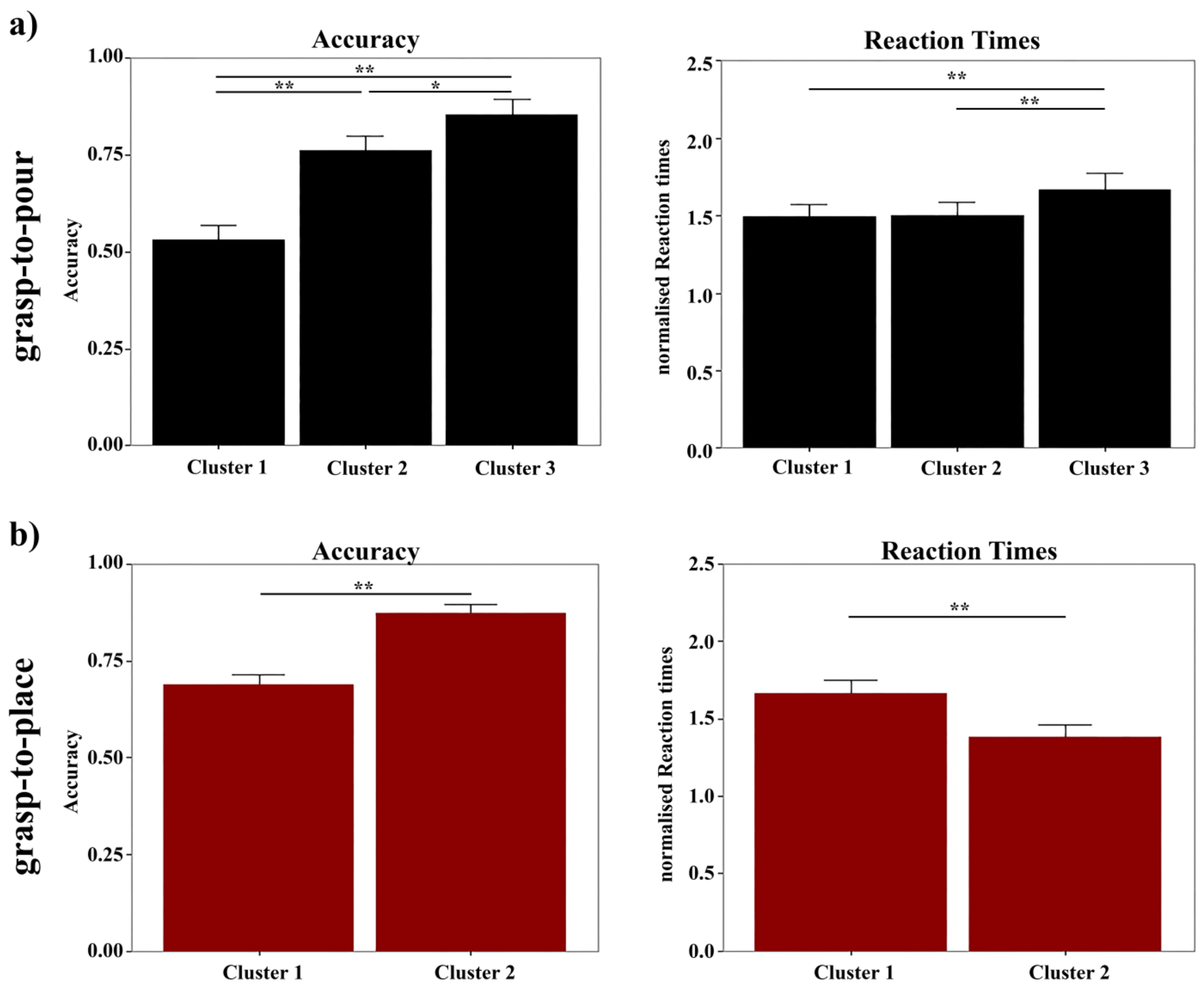

Figure S3. Accuracy and Reaction times for Experiment 3 (a) grasp-to-pour and (b) grasp-to-place movements. (* p <0.05, ** p <= 0.001)
